# A Translational Animal Model for Scar Compression Therapy Using an Automated Pressure Delivery System

**Published:** 2015-07-02

**Authors:** A. Alkhalil, S. Tejiram, T. E. Travis, N. J. Prindeze, B. C. Carney, L. T. Moffatt, L. S. Johnson, J. Ramella-Roman, J. W. Shupp

**Affiliations:** ^a^Firefighters’ Burn and Surgical Research Laboratory, MedStar Health Research Institute, Washington, DC; ^b^The Burn Center, Department of Surgery, MedStar Washington Hospital Center, Washington, DC; ^c^Department of Biomedical Engineering, Florida International University, Miami

**Keywords:** wound, pressure therapy, scar, animal model, red Duroc pig

## Abstract

**Background:** Pressure therapy has been used to prevent and treat hypertrophic scars following cutaneous injury despite the limited understanding of its mechanism of action and lack of established animal model to optimize its usage. **Objectives:** The aim of this work was to test and characterize a novel automated pressure delivery system designed to deliver steady and controllable pressure in a red Duroc swine hypertrophic scar model. **Methods:** Excisional wounds were created by dermatome on 6 red Duroc pigs and allowed to scar while assessed weekly via gross visual inspection, laser Doppler imaging, and biopsy. A portable novel automated pressure delivery system was mounted on developing scars (*n* = 6) for 2 weeks. **Results:** The device maintained a pressure range of 30 ± 4 mm Hg for more than 90% of the 2-week treatment period. Pressure readings outside this designated range were attributed to normal animal behavior and responses to healing progression. Gross scar examination by the Vancouver Scar Scale showed significant and sustained (>4 weeks) improvement in pressure-treated scars (*P* < .05). Histological examination of pressure-treated scars showed a significant decrease in dermal thickness compared with other groups (*P* < .05). Pressure-treated scars also showed increased perfusion by laser Doppler imaging during the treatment period compared with sham-treated and untreated scars (*P* < .05). Cellular quantification showed differential changes among treatment groups. **Conclusion:** These results illustrate the applications of this technology in hypertrophic scar Duroc swine model and the evaluation and optimization of pressure therapy in wound-healing and hypertrophic scar management.

Hypertrophic scar (HTS) is a common cutaneous complication following cutaneous trauma, surgery, infection, or burn. Scar tissue is visibly different from surrounding uninjured skin by measures of height, pliability, pigmentation, glossiness, and vascularity. Patients further experience severe itching, neuropathic pain, sleep disturbances, and impairment of daily activities.^1^ Cases of disfigurement or unpleasant aesthetics have led to psychological complications such as posttraumatic stress,^2^ loss of self-esteem,^3^ and stigmatization.^4^ Severe cases of scaring can cause contractures or disabling physical deformities.^5^ In developed countries, as many as 100 million people are estimated to acquire some form of scar following cutaneous injury. About 15% of these scars develop into further unaesthetic or debilitating conditions.^6^ A survey of major patient concerns after surgery showed that 91% of the patients favored better resolution for scar.^7^

The cellular and molecular mechanisms underlying impaired wound healing are poorly understood.^8^ Wounds resulting in HTSs exhibit longer epithelialization times, delayed and extended overlapping in wound-healing phases, abundant inflammatory mediator expression,^9^ and excessive accumulation of extracellular matrix (ECM) components such as collagen with modulated subtype proportions.

A variety of approaches are used in HTS management including surgical excision with or without grafting,^10^^-^12 intralesional interferon,^13^ topical and intralesional corticosteroids,^14^ intralesional bleomycin,^15^ silicone gel sheeting,^16^^,^^17^ and laser therapy.^18^ Mechanomodulatory strategies have proven effective in controlling incisional wound scarring.^19^ Pressure therapy has been used in HTS treatment,^20^ with varying degrees of success due to the limited understanding of its mechanism of action, nonstandardized application protocols, and lack of validated animal models.^8^^,^^21^

Developing a reproducible wound model is paramount to the study of scar physiology^22^ and assessing the efficacy of therapeutic intervention. While studying human tissue is most ideal, uncontrollable factors such as the injury depth, location, and patient compliance make this impractical. Research using Duroc pigs have increasingly documented similarities to human wound healing and scar formation by molecular, cellular, and gross measures. Unlike other animals such as guinea pigs, rabbits, or rats, red Duroc size is comparable to humans and offers flatter skin surfaces that make them the choice animal in large and producible wounds creation.

To date, there is no established tool offering precise pressure delivery or a validated animal model for assessing the effect of pressure in HTS therapy. Here, a novel automated pressure delivery system (APDS) capable of delivering an adjustable steady pressure was designed and tested in red Duroc scar model. The aim of this work was to evaluate the suitability of APDS in studying and characterizing the effect of pressure application in red Duroc swine as a model of HTS therapy under controlled conditions.

## MATERIALS AND METHODS

### Animal selection

Juvenile castrated male Duroc swine were handled according to facility standard operating procedures under the animal care and use program accredited by the Association for Assessment and Accreditation of Laboratory Animal Care International and Animal Welfare Assurance through the Public Health Service. All described animal work was reviewed and approved by the MedStar Health Research Institute's Institutional Animal Care and Use Committee.

### Experimental design

Six red Duroc pigs were used for wound creation with a Zimmer dermatome (Zimmer, Ltd, Swindon, United Kingdom). On each flank, a 4 x 4 in (10.16 x 10.16 cm) wound was excised over the lateral thorax to a partial-thickness depth of 0.060 in (0.030 in x 2 passes) or full-thickness depth of 0.090 in (0.0.030 in x 3 passes).

Wounds were dressed with Mepilex Ag (Monlylke, Gothenburg, Sweden) and changed regularly. Pain was managed by buprenorphine and fentanyl at the end of each procedure. Animals were examined at least twice daily to monitor pain, wound, or behavior changes. Animals were brought back weekly to the operating room, examined, and images and biopsy specimens collected. Punch biopsy specimens (3 mm) were taken pre- and postexcision at weekly assessments. Biopsy specimens were placed in formalin for histology or Allprotect Tissue Reagent (Qiagen, Valencia, Calif) for RNA and protein isolation.

After wound reepithelialization and scar development, an automated pressure delivery device was mounted on day 70 to scars. The treatment period lasted 2 weeks whereupon developed scars received pressure treatment (device/pressure at 30 mm Hg), sham treatment (device/no pressure), or no treatment at all (no device). Weekly assessments continued during and after pressure application.

### Automatic pressure delivery system

Briefly, the APDS consisted of a set of linear actuators for pressure delivery to underlying tissue and force-sensitive resistors for pressure measurements. Wireless communication allowed for pressure recording and feedback control to ensure accurate pressure delivery.^23^

### Surgical mounting of the APDS and pressure recording

Animals were sedated using a combination of ketamine and xylazine delivered intramuscularly, followed by intubation and general anesthesia delivery. Animals were maintained on isoflurane, placed on a warming blanket, and ventilated during examination or device mounting.

A Plexiglass base was secured to surrounding skin using MYO/WIRE II Sternotomy Suture (A&E Medical Corporation, Durham, NC), followed by APDS attachment. Protective padding was applied and reinforced using Delta fiberglass casting material (BSN Medical, Charlotte, NC). A custom-fitted neoprene vest was then placed to ensure further protection of the animal and device.^24^

Upon procedure completion, anesthesia was stopped and animals were brought back to the animal housing facility. Pressure recording started after animal recovery from anesthesia (Fig 2). Pressure boxes were removed temporarily after 1 week of pressure application (<3 hours) for scar assessment and biopsy specimen procurement and then permanently after 2 weeks of pressure application.

### Imaging

At each assessment, wounds or scars received standard digital imaging and Laser Doppler Imaging (LDI). The amount of perfusion was calculated by LDI to produce a mean perfusion unit. Moor LDI software (v5.3; Moor Instruments, Devon, United Kingdom) was used for image capture and analysis of mean perfusion units.

### Histology

Punch biopsy specimens were fixed in 10% formalin and embedded in paraffin. Paraffin blocks were sectioned (5 µm) and left to dry overnight. Slides were deparaffinized using xylene and dehydrated using an ethanol gradient. Staining was then performed using either hematoxylin and eosin (H&E) or the fluorescent dye DAPI. A Zeiss Axioimager microscope was used to view slides (Carl Zeiss, Oberkochen, Germany). Zeiss Zen Pro 2012 software (Carl Zeiss) was then used to capture digital images and conduct measurements.

### Assessment of skin thickness and cellularity

Sections' images were used for gross examination, skin layer measurements, and quantification of cells. Epidermal thickness was measured as the distance from the surface of the skin to the dermal-epidermal junction (µm). Dermal thickness was measured as the distance from the dermal-epidermal junction to the first identifiable sign of hypodermis (µm). Hypodermis was identified by the presence of lobules of fat or loose connective tissue compared with dermal layers.

Cell quantification was performed using ImageJ software (v1.48; NIH, Bethesda, MD) to produce a percent cellularity per high-powered field. Ten high-powered fields per section were used for cell quantification.

## RESULTS

### Reproducible HTS in red Duroc swine requires full-thickness wounds

**Figure 1:**
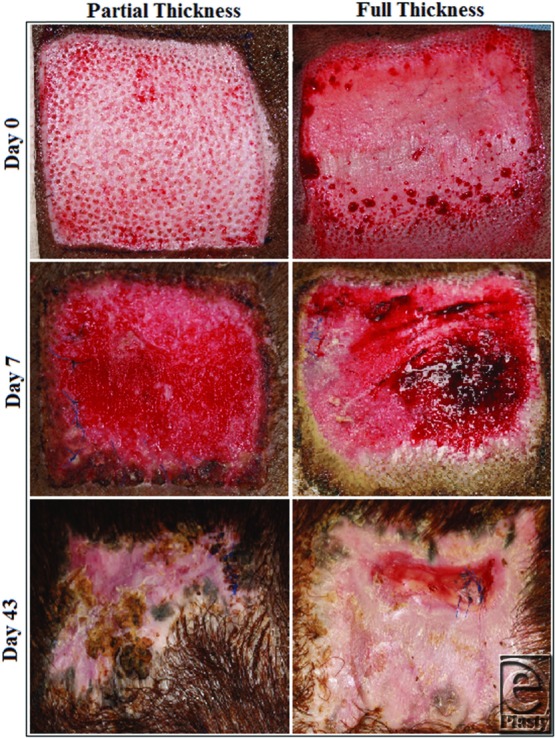
Comparison of partial-thickness and full-thickness wounds at different time points.

Six red Duroc swine had 4 x 4-in wounds created on their flanks with a dermatome. Two pigs received partial-thickness (0.06 in) wounds (*n* = 4) and 4 pigs received full-thickness wounds (*n* = 8). All partial-thickness wounds reepithelialized by day 7 and healed with no significant skin deformities (Fig 1, Table 1). Full-thickness wounds reepithelialized between 30 and 40 days (Fig 1).

Scars assessment using Vancouver Scar Scale (VSS) scores in all animals showed no significant differences prior to pressure therapy. Pressure-treated scars received lower VSS scores after 1 week of compression and significant decreases (*P* < .05) after 2 weeks compared with sham-treated and untreated scars (Tables 2 and 3). This effect was sustained on subsequent assessments following APDS removal (Fig 5).

### Gross examination of the effects of APDS mounting

**Figure 2:**
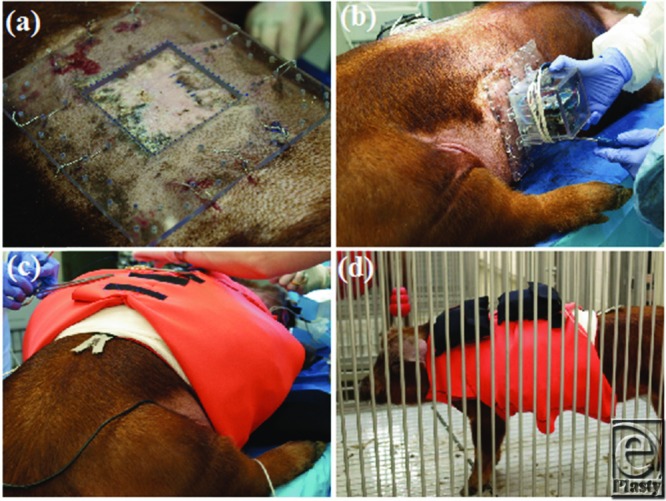
Mounting and protecting the pressure delivery system. Base mounting with sternal wire suture (a). automated pressure delivery system mounting to Plexiglass base (b). Neoprene vest application following dressings (c). Animal recovery and housing (d).

Wound assessments under anesthesia prior to APDS mounting were approximately 1 to 2 hours in duration. Mounting of the APDS device added approximately 2 additional hours. The most traumatic part of the APDS mounting process was securing the device base by sternal wire suture. At no point during the procedure deterioration in vital signs was noted. No signs of abnormal animal behavior or distress were noted after the procedure. Transient signs of inflammation were noted at suture entry and exit points, but no significant skin damage or signs of infection were observed (Fig 2).

Pressure boxes were removed once for no more than 2 hours to assess scar and collect biopsy specimens. Gross examination of sham-treated and untreated scars at days 70 and 84 postwounding showed similar scars, suggesting a negligible effect of the device mounting procedure on scar development.

### APDS performance and the effects of pressure on gross HTS characteristics

**Figure 3:**
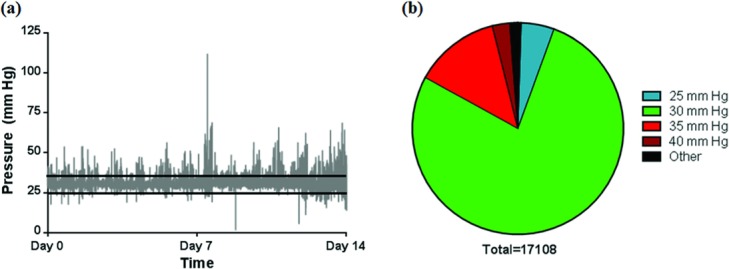
Analysis of pressure recordings from the pressure delivery system throughout the treatment period. Condensed plot of all pressure values recorded during the 14 days of pressure treatment (a) distribution of pressure values during pressure application (b). Note the cyclical distribution of the out-of-range pressure values that coincides with the number of days and activity periods of the animal (a). Distribution of abnormal pressure values showed higher frequency on the high side of pressure values. The frequency and intensity of abnormal pressure values show an increasing trend as the animal wound healing progresses and animals recover from automated pressure delivery system mounting procedure.

Pressure recordings for each device were analyzed to evaluate the performance of the APDS. The data showed that all APDS devices maintained a targeted pressure level of 30 ± 4 mm Hg for more than 90% of the total pressure application duration. The variations in pressure outside of the targeted pressure range accounted for about 9% to 10% of the total pressure application time (Fig 3). These variations mostly ranged between 22–26 and 34–38 mm Hg and were transient, underscoring the rapid response of the system in correcting pressure changes caused by animal activity (Table 4). Out-of-range pressure incidents were more frequent above the desired range than below it at a ratio of approximately 5:1, suggesting that animal behavior accounted for pressure fluctuations rather than mechanical APDS deficiencies. Pressure readings greater than the targeted pressure range accounted for only 8.8% of all pressure readings, and readings exceeding 40 mm Hg only accounted for 2% of all readings.

**Figure 4:**
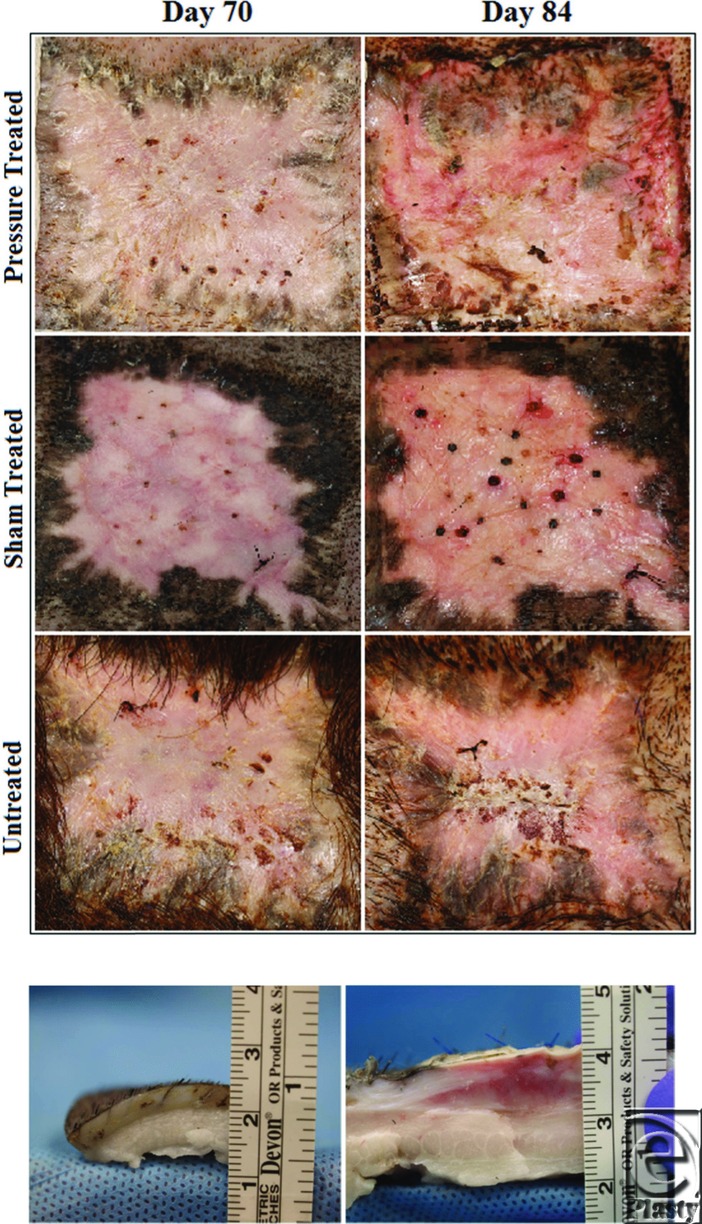
Direct gross comparison of scars before and after the treatment period (a). The difference in the thickness of the skin in a pressure-treated scar and sham-treated scar from the same animal after sacrifice (b).

**Figure 5:**
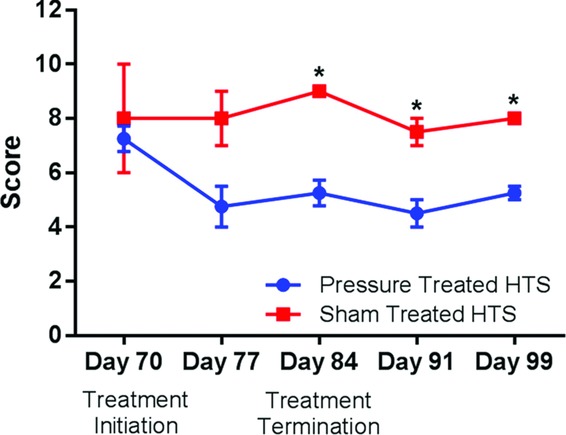
Changes in scar assessment using Vancouver Scar Scale scoring compared on the basis of treatment modality. *Significant differences were noted past day 77 between pressure-treated and the sham-treated scars.

Pressure-treated scars showed clear differences at day 84 postwounding compared with pretreated scar at day 70 or relative to sham-treated scars at day 84. These differences primarily encompassed the pliability, height, and vascularity parameters of the VSS (Tables 2 and 3). Pressure-treated scars consistently showed significantly lower total VSS values during the treatment period (*P* < .05; Tables 2 and 3, Fig 4). Continuous observation of scar development for 4 weeks after removal of pressure showed persistently lower VSS values in pressure-treated HTS compared with sham (Fig 5).

### Pressure delivered using the APDS induces changes in dermal thickness of HTS

**Figure 6:**
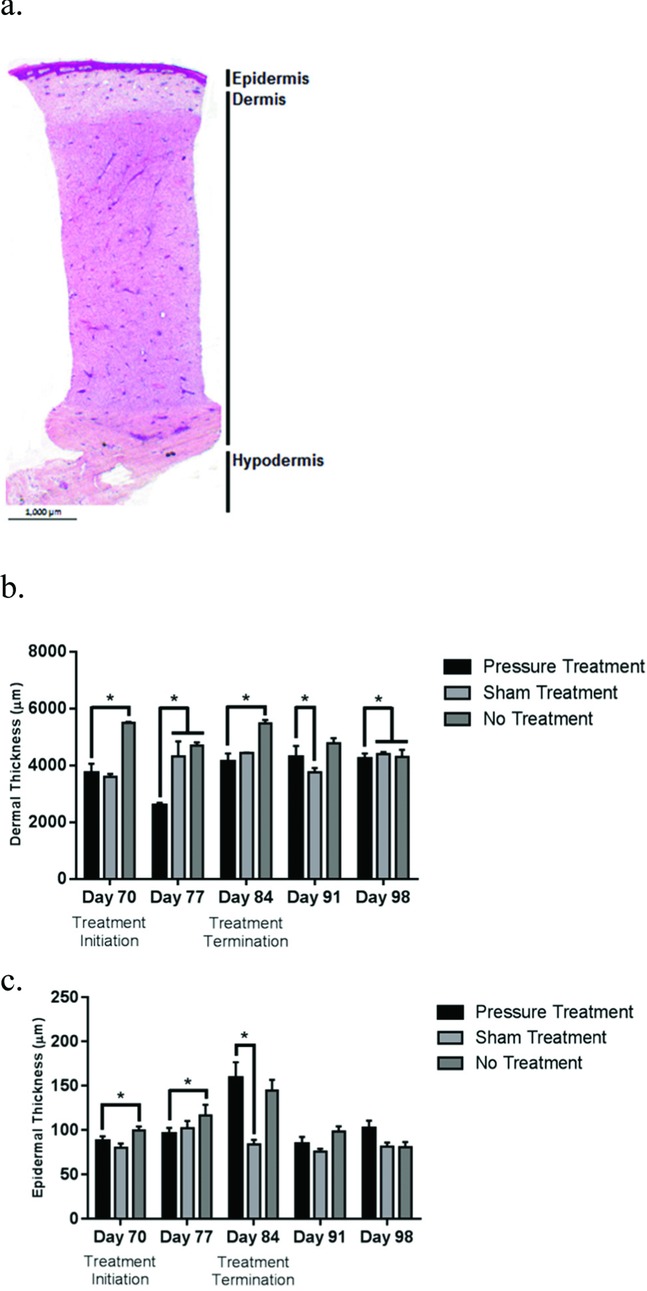
Comparison of changes in major skin layers after pressure application using H&E staining of sections. Representative image showing the main skin layer thickness (a). Thickness of the dermal (b) and epidermal (c) layers in biopsy specimens from pressure-treated, sham-treated, and untreated HTSs. HTS indicates hypertrophic scar. *Denotes statistical significance between treatment modalities.

Thickness of the epidermis and the dermis was quantified from H&E-stained sections (Fig 6 *a*) of scars. Comparative analysis revealed a steady trend of reduced dermal thickness in pressure-treated scars (Fig 6 *b*). Significant differences between sham and untreated scars were noted at days 70 and 84 postwounding (*P* < .05; Fig 6 *b*). A**s**sessment of changes in the epidermal layer showed nonspecific differences. These differences are probably due to heterogeneity of the epidermis across biopsy specimens (Fig 6 *c*). Note that the effect of applying pressure for 2 weeks caused persistent changes in the dermis for at least 4 weeks after APDS removal (Fig 6 *b*).

### Pressure delivered to HTS using the APDS induce changes in the behavior of HTS cells

**Figure 7:**
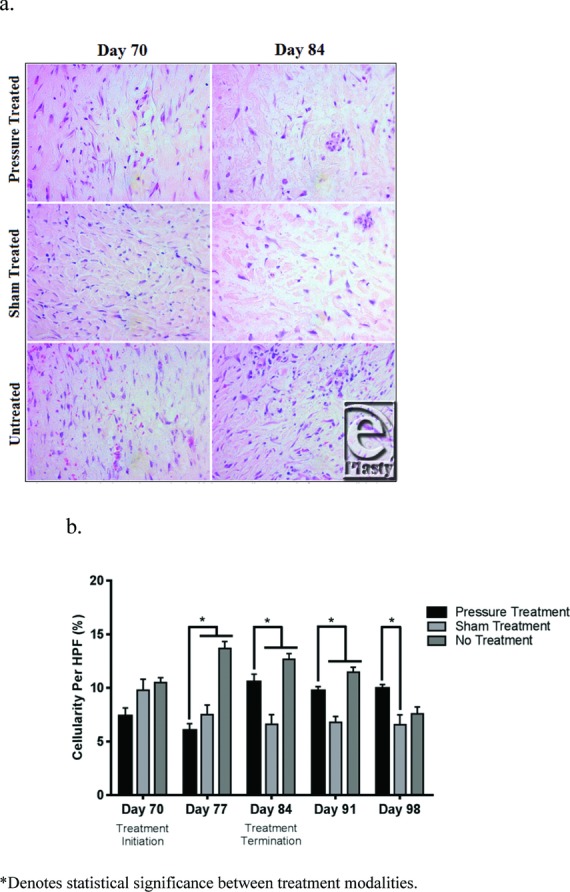
Sections of biopsy specimens from pressure, sham, and no treatment scars at days 70 and 84 (a). Average percent cellularity per HPF compared on the basis of treatment (b). HPF indicates high-powered field. *Denotes statistical significance between treatment modalities.

H&E-stained sections from pressure-treated, sham-treated, and untreated HTS biopsy specimens were examined under the microscope for quantifiable cellular changes during and after the treatment period (Fig 7 *a*) with results confirmed by DAPI fluorescent staining (not shown). Pressure-treated scars had a significant decrease in cellularity compared with both sham-treated and untreated scars 1 week into the treatment period (*P* < .05). However, cellularity increased significantly compared with its previous week as well as to sham-treated scars at day 84 and afterward (*P* < .05). Compared with untreated scars, pressure-treated scars had significantly lower cell counts after 1 week of treatment and up to 1 week after treatment (*P* < .05; Fig 7 *b*).

### Assessment of pressure-treated HTSs using LDI showed an increase in tissue perfusion relative to sham-treated HTS

**Figure 8:**
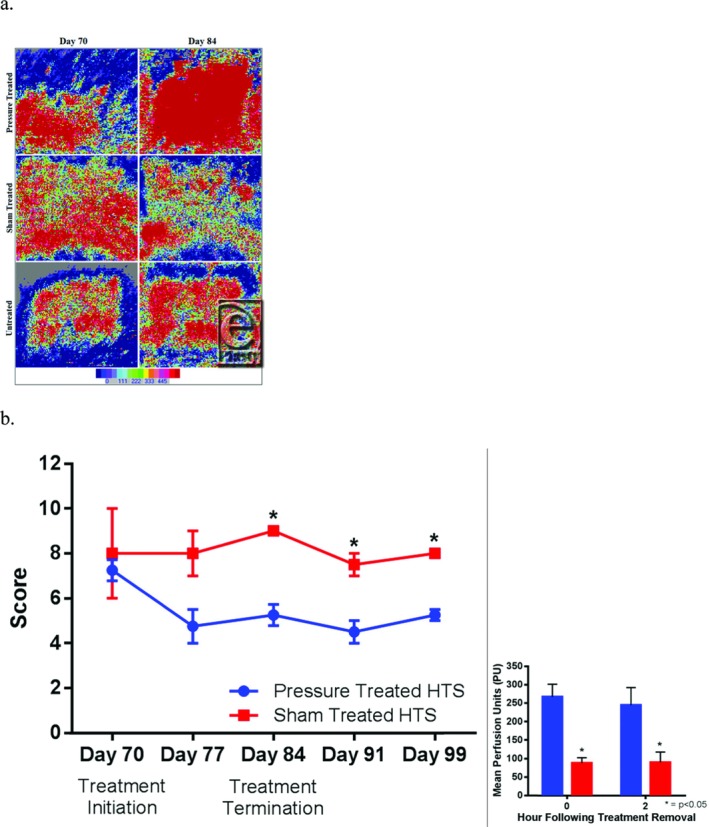
Comparison of laser Doppler imaging results between different treatment modalities during and after the compression period. Row images of pressure-treated, sham-treated, and untreated scars at days 70 and 84 (a), temporal perfusion changes of days 63 and 112 for pressure-treated and sham-treated scars (b). The inset shows perfusion 2 hours after removal of the automated pressure delivery system at day 84.

Evaluation of wound perfusion before and after APDS mounting using LDI showed differences between pressure-treated scar and other arms of the study. While sham-treated and untreated scar produced laser Doppler images suggestive of no significant change, pressure-treated scar laser Doppler images showed evidence of increased scar perfusion by day 84 (Fig 8 *a*). Further software-aided analysis confirmed a significant increase in scar perfusion (Fig 8 *b*) relative to sham-treated and untreated scars during the treatment period.

## DISCUSSION

Compression is commonly used in HTS therapy without clear understanding of its influence on scar pathophysiology. Varying garment elasticity over time and patient commitment are major hurdles to effectively study pressure therapy.^25^ The inability to reliably quantify pressure delivery has often resulted in suboptimal pressure application in scar treatment. A pressure delivery system capable of delivering controllable and precise pressure doses to scars 23 was engineered and tested in a red Duroc model of HTS.^22^^,^^26^ The system featured wireless real-time recording of pressure and minimal restriction of animal mobility. This enabled unimpeded characterization of device function that revealed additional information about the relation of animal behavior and delivery of steady pressure.

A standard protocol to generate reproducible HTSs was critical to the evaluation of the APDS. Deeper full-thickness wounds were required for HTS in this red Duroc model. The newly generated tissue was typical of an HTS such that it was raised, contracted, swollen, and less pliable than the uninjured skin.

The summative decrease in epidermal and dermal layer thickness was less than the overall decrease in scar height following pressure application, suggesting that subdermal layers must be considered in determining the effect of pressure on HTS and skin thickness dynamics.

The decrease in height may result from loss of local fluid, cells, and/or ECM. The immediate effect of pressure application is reduction in blood flow, which will cause marginal reduction in scar volume. However, the final decrease in scar height and the persistence of changes in scar tissue following removal of pressure suggest the involvement of more complex mechanisms.^27^^,^^28^

Hypoxia has been described to induce changes in cell proliferation, differentiation, and survival in different cell lines.^29^ Similarly, mild hypoxic conditions correlated with modulation of cell metabolism and secretome.^30^ It has been proposed that the hypoxic conditions induced by pressure result in cellular changes that ultimately reduce collagen deposition and decrease scaring.^31^ While this hypothesis might be valid, further investigations are still needed for direct evidence and specific mechanisms, given the variety of cell and tissue types used to generate these results and the diverse and dynamic cellular content of scar.

The significant variations in total cell count and the associated decrease in scar height after pressure application observed in this work suggest changes in cell behavior and/or the homeostatic balance of cell types in scars under pressure. Cellularity changes reported in treatment groups testify to the complex interactions involved. Changes in the total cellularity of scars undergoing compression are influenced by compounded variables such as the described decreases in apoptosis rates in HTSs^32^ and increased rates of cell apoptosis and upregulation of IL-1β and TNF-α described in vitro in response to compression (35 mm Hg/24 h).^33^ Shifts in cell-type balance in pressure-treated scars are possible. Such changes would not be distinguished in the cell counting method used here. Changes in ECM are a natural correlate of shifts in cell count, type, and activity. Further work is underway to identify and characterize changes in cell activities and cell types upon pressure application on scars.

The neovascularization enhancement is consistent with reports of increased vasculogenesis under hypoxic conditions. 34 Interestingly, this also associates with changes in cellular secretome, which could be a way for ECM deposition modulation. The increased perfusion in pressure-treated scars noticed after removal of the APDS might be different from perfusion levels under compression. This perfusion increase change persisted for at least 2 hours after the APDS is removed and went back to levels similar to that of sham-treated scars after 1 week. This suggests that perfusion changes directly related to pressure and that the mechanisms regulating perfusion homeostasis are preserved in HTS.

## Figures and Tables

**Table 1:** Wounds dimensions and progression to scar in used animals

Animal sequential #	Animal side	Wound* depth, in	Time to reepithelialization, d	Scar formed†
1	Left	0.06	≥7	No
1	Right	0.06	≥7	No
2	Left	0.06	≥7	No
2	Right	0.06	≥7	No
3	Left	0.09	42	Yes
3	Right	0.09	56	Yes
4	Left	0.09	56	Yes
4	Right	0.09	56	Yes
5	Left	0.09	42	Yes
5	Right	0.09	35	Yes
6	Left	0.09	35	Yes
6	Right	0.09	35	Yes

*All wounds had the same 4 × 4 in (W × L) dimensions.

†All full-thickness wounds resulted in scars by day 63.

**Table 2:** Wound assessment using VSS scores*

Animal sequential #	Study day	Animal side	Treatment	Vascularity	Pigmentation	Pliability	Height	Total
3	70	Left	NA	1	2	3	2	8
3	70	right	NA	1	2	3	2	8
3	84	Left	Pressure	1	2	3	0	6
3	84	Right	Pressure	1	2	3	0	6
5	70	Left	NA	1	2	2	1	6
5	70	right	NA	1	2	2	1	6
5	84	Left	Sham	1	2	3	3	9
5	84	Right	Pressure	1.5	2	1	0	4.5
6	70	Left	NA	3	2	2	3	10
6	70	right	NA	1	2	2	2	7
6	84	Left	Sham	1	2	3	3	9
6	84	Right	Pressure	1	2	1	0	4

*Score of each wound from 3 animals receiving assorted treatments at days 70 to 84.

VSS indicates Vancouver Scar Scale; NA, not applicable.

**Table 3:** The mean VSS score during the treatment period grouped by treatment modality*

Treatment	Day	Vascularity	Pigmentation	Pliability	Height	*VSS score*
Pressure	70	1	2	2.5	1.8	*7.3*
	84	1.1	2	2	0	*5.1*
Sham	70	2	2	2	2	*8*
	84	1	2	3	3	*9*

*Untreated scar (animal 4) was not assessed by the VSS score during the pressure application period. Data represent the average score from 2 animals.

VSS indicates Vancouver Scar Scale.

**Table 4:** Summary of out-of-range pressure data analysis

	Days after compression therapy													
Criteria	1	2	3	4	5	6	7	8	9	10	11	12	13	14
Total # of events	114	177	75	78	112	167	105	88	93	104	115	156	445	207
# Events >34 mm Hg	107	167	71	68	103	159	103	65	90	102	114	146	262	141
Upper to lower out-of-range ratio	15.29	16.70	17.75	6.80	11.44	19.88	51.50	2.83	30.00	51.00	114.00	14.60	1.43	2.14
Avg event length, min	1.044	1.034	1.04	1	1.018	1.006	1.029	1	1.054	1.029	1.017	1.032	1.063	1.091
Total time, min	119	183	78	78	114	168	108	88	98	107	117	161	473	227
Max event length, min	2	2	2	1	2	2	2	1	3	2	2	4	4	4
Max pressure, mm Hg	54.06	54.23	48.32	52.91	47.68	56.15	55.14	112.1	50.5	56.72	53.94	66.02	62.55	68.83
